# S173. GREY MATTER VOLUME DEFFICITS IN PATIENTS WITH A FIRST EPISODE NON-AFFECTIVE PSYCHOSIS AND SUICIDE RELATED BEHAVIOUR

**DOI:** 10.1093/schbul/sby018.960

**Published:** 2018-04-01

**Authors:** Diana Tordesillas-Gutiérrez, Manuel Canal-Rivero, Esther Setien-Suero, Rosa Ayesa-Arriola, Manuel Delgado-Alvarado, Benedicto Crespo-Facorro

**Affiliations:** 1 Neuroimaging Unit, Technological Facilities, Valdecilla Biomedical Research Institute IDIVAL; 2 Neuroimage Unit, Technological Facilities, IDIVAL; 3 University Hospital Marques de Valdecilla -IDIVAL; University of Cantabria, CIBERSAM

## Abstract

**Background:**

Suicide represents the main cause of premature dead in first episode psychosis (FEP) patients. However, our understanding of suicidal behaviour in this population is limited. During the last decade, several works have related suicidal behaviour in FEP patients with structural abnormalities in frontal and temporal areas as well as specific structures such as hippocampus, insula and amygdala. The main aim of this work was to analyse the possible structural brain abnormalities associated with suicide-related-behaviour in a large sample of FEP patients.

**Methods:**

We use a voxel-based morphometry (VBM) analysis in 146 FEP individuals: 24 FEP with and 122 without suicidal behaviour. All images were taken in the same 3T Phillips scanner. The CAT 12 toolbox, which is implemented in SPM12 was used for VBM analysis of the data. A two-sample t-test was set with sex, age, handedness, total intracraneal volume and global disability score as nuisance covariables. We applied threshold-free cluster enhancement (TFCE) with 5000 permutations and corrected for multiple comparisons (FWE) at p<0.05.

**Results:**

A gradual reduction of grey matter volume related to presence of suicide-related-behaviour was found in frontal area, specifically in superior frontal gyrus, middle frontal gyrus, precentral gyrus, inferior frontal gyrus and orbital gyrus. In addition, significant reduction was found in middle temporal gyrus as well as in posterior cingulate gyrus and precuneus.

**Discussion:**

Our results are in line with previous works which related suicidal behaviours with reduced frontal regions. Frontal areas are involved in: i) cognitive analysis; ii) foresight and weighing consequences of behaviour; iii) considering future and making predictions; iv) impulse control; v) delaying gratification; vi) inhibiting inappropriate behaviour; vii) initiating appropriate behaviour. Reestructuraria esta frase asi: On the other hand, precuneus is involved in: i) episode memories; ii) reflective self-awareness; iii) executive function; and iv) it is activated during judgements. Finally, cingulate gyrus has been strongly associated with emotional responses to pain, regulation of aggressive behaviour and decision making. Finally, middle temporal gyrus appears to play an important role in retrieving semantic information.

This study provides some insights about brain abnormalities associated with suicide-related-behaviours in FEP patients. In particular, the areas reported in this study are related with important functions such as impulsivity, emotional processing information, responses to pain and aggressiveness which are strongly associated with suicide-related-behaviours. Further studies are necessary to replicate the relevance of these structures in suicidal behaviour in FEP patients.

